# Test-Retest Reliability of fMRI During an Emotion Processing Task: Investigating the Impact of Analytical Approaches on ICC Values

**DOI:** 10.3389/fnimg.2022.859792

**Published:** 2022-05-10

**Authors:** Mickela Heilicher, Kevin M. Crombie, Josh M. Cisler

**Affiliations:** ^1^Mental Health and Incarceration Laboratory, Psychiatry Department, School of Medicine and Public Health, University of Wisconsin-Madison, Madison, WI, United States; ^2^Neurocircuitry of Trauma and PTSD Laboratory, Department of Psychiatry and Behavioral Sciences, Dell Medical School, The University of Texas at Austin, Austin, TX, United States; ^3^Department of Psychiatry and Behavioral Sciences, Dell Medical School, Institute for Early Life Adversity Research, The University of Texas at Austin, Austin, TX, United States

**Keywords:** reliability, fMRI, independent component analysis, voxelwise, contrast, intraclass correlation coefficient

## Abstract

Test-retest reliability of fMRI is often assessed using the intraclass correlation coefficient (ICC), a numerical representation of reliability. Reports of low reliability at the individual level may be attributed to analytical approaches and inherent bias/error in the measures used to calculate ICC. It is unclear whether low reliability at the individual level is related to methodological decisions or if fMRI is inherently unreliable. The purpose of this study was to investigate methodological considerations when calculating ICC to improve understanding of fMRI reliability. fMRI data were collected from adolescent females (*N* = 23) at pre- and post-cognitive behavioral therapy. Participants completed an emotion processing task during fMRI. We calculated ICC values using contrasts and β coefficients separately from voxelwise and network (ICA) analyses of the task-based fMRI data. For both voxelwise analysis and ICA, ICC values were higher when calculated using β coefficients. This work provides support for the use of β coefficients over contrasts when assessing reliability of fMRI, and the use of contrasts may underlie low reliability estimates reported in the existing literature. Continued research in this area is warranted to establish fMRI as a reliable measure to draw conclusions and utilize fMRI in clinical settings.

## Introduction

Functional magnetic resonance imaging (fMRI) is commonly utilized to investigate neural activity related to behavior; therefore, decent reliability of fMRI as a measurement is crucial for drawing conclusions from imaging research. Reliability is often assessed via test-retest reliability procedures, wherein data is collected via a particular measurement (i.e., fMRI) at two timepoints to examine the extent to which results are comparable between time one and time two. Thus, reliability reflects the degree to which a measure yields consistent results under similar circumstances (Elliott et al., 2020). There are numerous ways to measure test-retest reliability such as, but not limited to, Pearson's correlation, the Intraclass Correlation Coefficient (ICC), the Kendall coefficient of concordance, and the Dice coefficient (Noble et al., 2019). The ICC is most commonly used to assess test-retest reliability (Koo and Li, 2016). ICC is a numerical representation of the degree of correlation and agreement between two observations. ICC values are interpreted on a scale with anchors at poor (<0.4), fair (between 0.4 and 0.59), good (between 0.6 and 0.74), and excellent [>0.75; (Cicchetti, 1993)].

In order to confidently relate neural activity to psychological constructs of interest and apply research findings to clinical settings, such as establishing neural biomarkers of certain disorders, the observed neural activity from fMRI must be reliably stable. The overall degree of fMRI test-retest reliability can vary between task types (Holiga et al., 2018), specific conditions of a task and the contrasts of interest (Raemaekers et al., 2007; Fröhner et al., 2019; Heckendorf et al., 2019; McDermott et al., 2020), as well as the brain region of interest [ROI; (Plichta et al., 2012; Li et al., 2020; Morales et al., 2020; Korucuoglu et al., 2021)]. Low reliability in imaging research limits inferences that relate individual difference measures to fMRI activation (Zeynep Enkavi et al., 2019). However, common trends in the literature emerge when considering (1) the relationship between activation and degree of reliability, and (2) reliability of group-level activation vs. individual-level activation.

For instance, regions with greater activation or significantly activated voxels to the task at both time points show greater test-retest reliability (Brandt et al., 2013; Bossier et al., 2020). Several studies have found a positive relationship between neural activation and ICC values during memory (Bennett and Miller, 2013), a response inhibition (Korucuoglu et al., 2021), and risk taking behavior tasks (Li et al., 2020). Another study found that voxels with greater activation at the group level had a higher probability of greater ICC values (Caceres et al., 2009). Relatedly, given the signal to noise ratio of different brain regions and structures, ICC values are typically found in cortical compared to subcortical regions (Korucuoglu et al., 2020). Therefore, signal strength likely plays a role in varying levels of fMRI reliability.

Across various tasks, measures of reliability, such as ICC, are greater at the group level than the individual level. This finding has been demonstrated during memory encoding tasks (Brandt et al., 2013; Holiga et al., 2018; Bossier et al., 2020), an intertemporal choice task (Fröhner et al., 2019), emotional face tasks (Plichta et al., 2012; Holiga et al., 2018; McDermott et al., 2020), an antisaccade paradigm (Raemaekers et al., 2007), reward-related tasks (Plichta et al., 2012; Holiga et al., 2018), N-back working memory tasks (Plichta et al., 2012; Holiga et al., 2018), a theory of mind task, and a response inhibition task (Holiga et al., 2018). It is currently unclear whether low reliability at the individual level is related to methodologies (e.g., measure of neural activation, imaging analysis) being used to calculate reliability or if fMRI is an inherently unreliable measure of neural activity. Therefore, the aim of this study is to investigate analytical approaches and methodological considerations when calculating reliability of fMRI using ICC. We focused on examining the value used to quantify neural activation and the type of imaging analysis (e.g., voxel-wise vs. network level).

Most of the current work concerning reliability of fMRI calculates reliability measures using difference scores (i.e., contrasts) reflecting changes in neural activation between two task conditions rather than a direct measure of functional activation [e.g., β coefficient from first-level general linear model (GLM)], which is likely to underestimate the true reliability of task-based fMRI. That is, the statistical literature has reported for some time that difference scores are inherently biased and unreliable, which is evident by their lack of use in clinical research and the transition to regression based analyses (Cronbach and Furby, 1970; Vickers, 2001). For instance, in an assessment of task and survey reliability, Zeynep Enkavi et al. (2019) found that task reliability was poor at the individual level, potentially attributable to the use of difference scores which have low between-subject variance.

High between-subject variance of a dependent measure contributes to greater reliability because the measure better reflects differences between subjects in a sample. Difference scores, however, have low between subject variance (Zeynep Enkavi et al., 2019), which, when used in reliability calculations, results in low reliability estimates. Therefore, a single measure, rather than a difference score such as a contrast, is better suited for assessing individual level reliability. Furthermore, as a result of being collected at the same time, the two measures subtracted in a difference score are highly correlated (Cronbach and Furby, 1970). A correlation between two variables subtracted from one another results in a high degree of error (Cronbach and Furby, 1970), and ultimately, lower reliability estimates. This concept has been exemplified in imaging literature in which a study found that amygdala activation during different conditions (faces vs. shapes in a matching task) was highly correlated (Infantolino et al., 2018). Therefore, we posit that low levels of individual level reliability can be explained by the use of contrasts in ICC calculations. We argue that ICC calculation should use a direct measure of functional activation (i.e., β coefficients derived from the general linear model). One prior investigation has shown improved test-retest reliability using beta coefficients, although this was done with data collected during a Balloon Analog Risk Taking Task (Korucuoglu et al., 2020), which highlights the need for investigations that assess other outcomes (e.g., emotion processing).

There are several advantages and disadvantages to voxel-wise vs. network level analysis of brain data. The current fMRI test-retest reliability research has primarily employed voxel-wise analyses. With a whole brain approach or ROI approach, which makes a priori assumptions about brain activation to a particular task or task condition, voxel-wise analyses characterize how specific regions respond under certain conditions (Cole et al., 2010). Additionally, a voxel-wise approach conducts a statistical test at each voxel at the whole brain level or at each ROI, which results in relatively low statistical power due to the high number of statistical tests run. On the other hand, network analyses, such as through independent component analysis (ICA), identify distinct networks of brain regions that are co-actively engaged throughout a task. Investigations of reliability using various network analyses, such as ICA (Guo et al., 2012; Blautzik et al., 2013) and connectivity network mapping (Chou et al., 2012) have found most large-scale networks to be highly reliable (see Noble et al., 2019) for a comprehensive review of test-retest reliability of functional connectivity). As a data-driven approach, network analyses can account for signals unknown a priori and, ICA specifically serves to isolate neural networks at rest or while engaged in a task (Ross and Cisler, 2020). Therefore, we chose to further assess whether either method, voxelwise analysis or ICA, yields higher ICC values.

The purpose of this study was to investigate methodological considerations when assessing test-retest reliability of fMRI using ICC. Using data from a sample of adolescent girls with Posttraumatic Stress Disorder (PTSD) prior to and following trauma-focused cognitive behavior therapy, we calculated ICC values using β coefficients and contrasts separately taken from a whole-brain voxel-wise analysis and network level analysis using ICA. We predict that, (1) given the high degree of error that results from ICC calculations using contrasts, ICC values will be higher using β coefficients for both the voxel-wise analysis and ICA; (2) ICC values will be higher for the ICA analysis compared to the voxelwise analysis; (3) there will be a positive relationship between ICC values and neural activation; and 4) higher ICC values will be concentrated in cortical, rather than subcortical, regions. It should be noted that using data at pre- and post-treatment is likely to provide more conservative tests of reliability, due to treatment-inducing changes in the neurocircuitry being measured. Therefore, it might be expected that we find lower absolute values of reliability (i.e., an overly conservative test of reliability); however, the within subject comparisons of β coefficients vs. contrasts and voxelwise vs. network should nonetheless be valid and informative regarding the impact of analytical approaches and methodological decisions on reliability estimates.

## Materials and Methods

The work described in this manuscript has been carried out in accordance with The Code of the Ethics of the World Medical Associations (Declaration of Helsinski) for experiments involving humans, and all subjects completed informed consent. The analyses included in the current manuscript are independent of previously reported findings, in which experience of assault was associated with greater reactivity of the salience network during the facial emotion processing task (Cisler et al., 2015, 2019).

### Subjects

Recruitment of participants and data collection took place at the University of Arkansas in Little Rock, AR. Participants consisted of 23 adolescents assigned female at birth, aged 11–17, undergoing trauma-focused cognitive behavior therapy for trauma-related symptoms following assaultive violence exposure. Assaultive violence exposure was operationalized as a direct experience of physical or sexual assault that the girl could remember. All participants met DSM-IV criteria for a PTSD diagnosis. Exclusion criteria included any histories of psychotic symptoms, neurocognitive disorders, presence of a developmental disorder, major medical disorders, MRI contradictions (e.g., non-removable metal), pregnancy, and history of traumatic brain injury. All study procedures were approved by the Institutional Review Board at the University of Arkansas for Medical Sciences (UAMS), and all methods were carried out in accordance with relevant guidelines and regulations. Twenty-two participants had pre- and post-treatment scans; one participant only had pre-treatment scans.

### Assessments

PTSD symptoms were assessed by a trained research staff using the UCLA PTSD Reaction Index (Steinber et al., 2004). The presence of mental health disorders was either assessed using the Mini-International Interview for Children and Adolescents (MINI-Kid; Sheehan et al., 2010) or Kiddie Schedule for Affective Disorders and Schizophrenia (K-SADS, Kaufman et al., 1997). Trained research staff administered the National Survey of Adolescents (NSA; Kilpatrick et al., 2000) trauma section, in order to assess trauma histories. The NSA is a structured interview that includes questions regarding exposure to physical abuse, sexual assault, witness domestic violence, witnessed community violence, and a various other stressors and traumatic events. Participants were also asked to complete several self-report measures containing questions regarding childhood maltreatment, depression symptoms, emotion regulation abilities, and PTSD symptoms. The self-report measures included the Childhood Trauma Questionnaire (CTQ; Bernstein et al., 1994), the Difficulties in Emotion Regulation Scale (DERS; Gratz and Roemer, 2004), the UCLA-PTSD Reaction Index (Steinberg et al., 2013), and the Short Mood and Feelings Questionnaire (SMFQ; Sharp et al., 2006).

### Treatment

Treatment was administered by a graduate or post-doctoral level therapist using a standardized protocol, and consisted of 12 trauma-focused cognitive behavioral therapy sessions (Cisler et al., 2016). Thirty-one participants were recruited, 22 of which had at least one usable scan for imaging analyses and 21 of which were used in reliability analyses.

### Face Emotion Processing Task

The emotion processing task is widely used in psychopathology research (Rauch et al., 2000; Williams et al., 2006; Brunetti et al., 2010) and data from this sample has previously been published by our group (Cisler et al., 2015, 2018). While in the MRI, participants viewed facial stimuli and made button presses indicating decisions concerning the sex of the face. The faces either exhibited a neutral or fearful expression (valence), presented overtly or covertly (duration) in alternating blocks. Overtly presented faces were presented for 500 ms; covertly presented faces were presented for 33 ms immediately followed by a neutral facial expression mask of the same actor in the covert image. Participants completed two runs of the task (roughly 8 min each), in which each block was presented 5 times. Contrasts of interest included covert fear vs. covert neutral, overt fear vs. overt neutral, covert fear vs. overt neutral, overt fear vs. overt neutral, and all fear vs. all neutral blocks. The task was administered before and after treatment with an approximate test-retest interval of 12-weeks (i.e., time to complete 12-week treatment). For additional information on task design see our groups previous work (Cisler et al., 2015).

### MRI Data Acquisition and Pre-processing

fMRI data were acquired on a Philips Achieva 3T X-series scanner using a 32-channel headcoil. T1-weighted anatomic images were acquired with a MP-RAGE sequence (matrix = 192 × 192, 160 sagittal slices, TR/TE/FA = 7.5/3.7/9°, FOV = 256, 256, 160, final resolution = 1 × 1 × 1 mm resolution). Echo planar imaging sequences were used to collect the functional images using the following sequence parameters: TR/TE/FA = 2,000 ms/30 ms/90°, FOV = 240 × 240 mm, matrix = 80 × 80, 37 axial slices (parallel to AC–PC plane to minimize OFC signal artifact), slice thickness = 2.5 mm, and final resolution of 3 × 3 × 3 mm.

Image preprocessing was completed using AFNI software and followed standard steps. In the following order, images underwent despiking, slice timing correction, deobliquing, motion correction using rigid body alignment, alignment to participant's normalized anatomical images, spatial smoothing using a 8 mm FWHM Gaussian filter (AFNIs 3dBlurToFWHM that estimates the amount of smoothing to add to each dataset to result in the desired level of final smoothing), detrending, high frequency (128 s) bandpass filtering, and rescaling into percent signal change. Images were normalized using the MNI 152 template brain. Following recommendations (Power et al., 2014; Siegel et al., 2014), we corrected for head motion related signal artifacts by using motion regressors derived from Volterra expansion, consisting of [R(t) R(t)^2^ R(t−1) R(t−1)^2^], where R refers to each of the 6 motion parameters, and separate regressors for mean signal in the CSF and WM. This step was implemented directly after motion correction and normalization of the EPI images in the image preprocessing stream. Additionally, we censored TRs from the first-level GLMs based on threshold of framewise displacement (FD) > 0.4. FD refers to the sum of the absolute value of temporal differences across the 6 motion parameters; thus, a cut-off of 0.4 results in censoring TRs where the participant moved, in total across the 6 parameters, more than ~0.4 mm plus the immediately following TR (to account for delayed effects of motion artifact). Additionally, we censored isolated TRs where the preceding and following TRs were censored, and we censored entire runs if more than 50% of TRs within that run were censored. The mean percent of TRs at time one was 83% (SD = 0.2) and at time two was 76% (SD = 0.2). Participants with more than one run removed were removed from analyses. This led to the removal of 2 participants.

### Data Analysis

#### Voxel-Wise Analysis

First-level analyses consisted of standard voxel-wise GLMs, in which a design matrix consisted of predictors for each task block type (overt fear, overt neutral, cover fear, covert neutral; see Cisler et al. (2015)). This resulted in four β coefficients, corresponding to the task design, for each voxel for each participant at pre- and post-treatment. Second-level analyses consisted of voxelwise linear mixed effects models (LMEMs), implemented in Matlab using custom scripts. One participant only had a pre-treatment scan and was thus excluded from the voxelwise analyses (*N* = 22). The task was modeled with a factorial design and included additional covariates nested within subjects as a random effect: activity ~ valence (neutral vs. fear) × duration (overt vs. covert) + age + IQ + head motion + (1|sub). Cluster-level thresholding (Eklund et al., 2016) controlled for voxel-wise comparisons using an uncorrected *p* < 0.001 and cluster size k ≥ 17. We used cluster-level thresholding as implemented in AFNI software. First, we estimated the actual amount of smoothing in the data using 3dFWHMx with the ACF option to account for non-Gaussian shaped smoothing functions. Second, using the actual amount of smoothing in the data, we used 3dClustSim to calculate the minimum cluster-size needed for a corrected *p* < 0.05 given a voxelwise *p* < 0.001 threshold, the actual smoothing of the data, and the gray matter mask created for these data. This analysis identified a cluster size of 17 voxels. Voxelwise analyses were constrained within a sample-specific gray matter mask consisting of 46,976 voxels. Voxelwise activation results are reported for mean activation across the emotion task and the valence effect of the LMEM.

#### Independent Components Analysis

**Figure 1:**
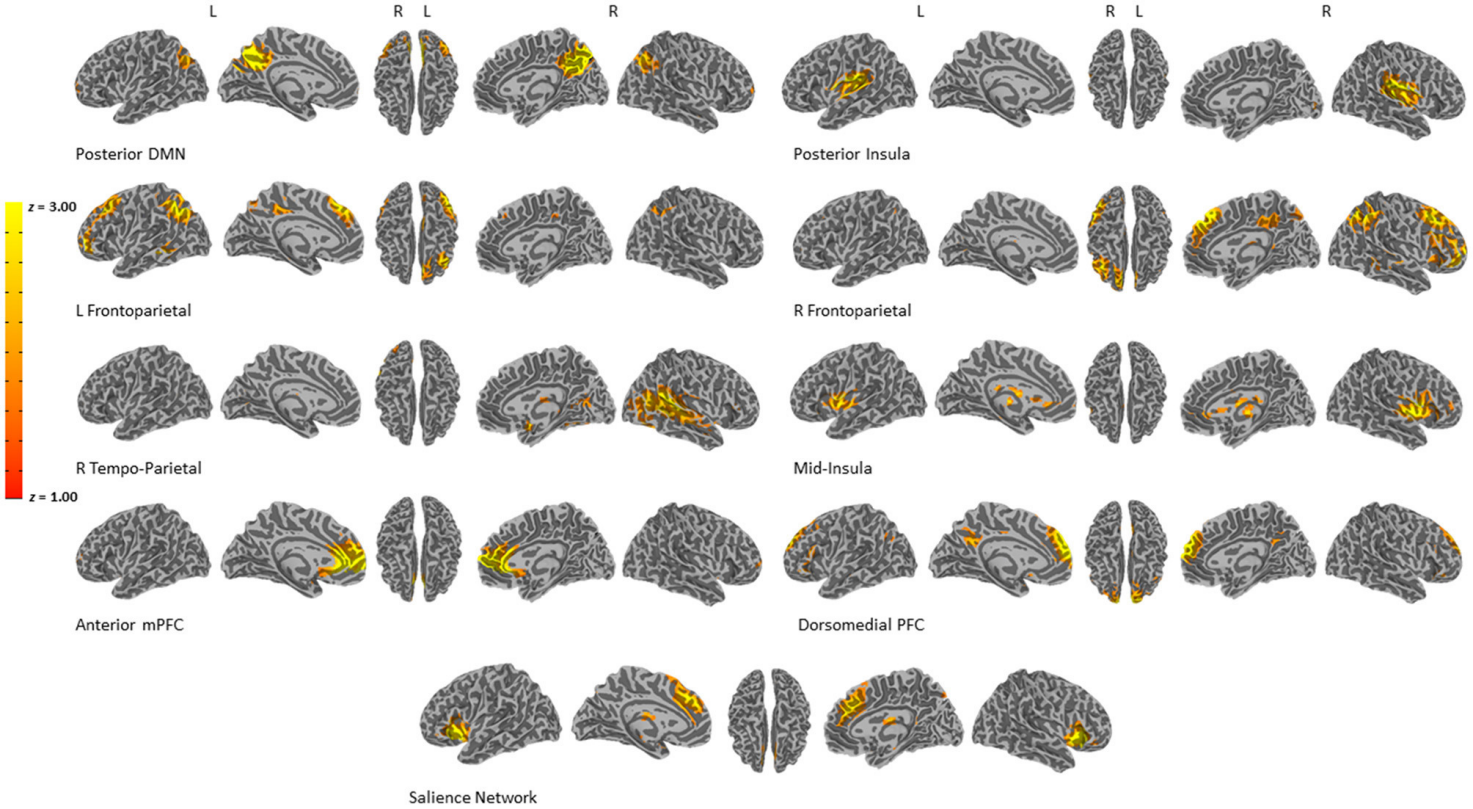
Results of the ICA analysis. Nine components of interest were chosen for reliability analyses.

We used GIFT in Matlab to implement spatial ICA to identify large-scale networks comprised of temporally coactive voxels, with a model order of 50 components (Calhoun et al., 2002). Task data from all task runs, from both pre- and post-treatment were combined for one ICA analysis. To improve precision of the ICA we used all available data (i.e., using all 23 participants' data regardless of whether only pre-treatment data was available). Of the 50 networks, we identified nine networks that were either canonical networks (Menon, 2011), responsive to the task (significant main effect of valence or duration, or significant valence by duration interaction at *p* < 0.05) or theoretically related to emotion processing and PTSD [i.e., excluding 41 networks that represented cerebral spinal fluid (CSF), artifact due to head motion, or networks that were non-responsive to the task or were of non-interest, such as motor or visual cortex; see Figure 1]. First-level analyses of the ICA timecourses used identical design matrices as the voxelwise analyses described above, resulting in β coefficients for all 11 components for each condition, per participant. Second-level analyses consisted of identical LMEMs as described for the voxelwise analyses. The LMEMs were conducted on each of the 11 components. We only considered components that were significantly engaged in the task (i.e., significant main or interacting effects of the task) after controlling for multiple comparisons with Bonferroni correction. We also included networks that were clearly theoretically related to the task and population. This resulted in 9 components that were carried forward to reliability analyses.

#### Intraclass Correlation Coefficient Analysis

To investigate test-retest reliability we calculated ICC (two-way mixed effects, single measurement, absolute agreement; ICC(A,1) in McGaw and Wong convention) using measurements of task-based functional brain activity collected at pre- and post-treatment for 22 participants. In order to investigate methodological considerations when assessing reliability with ICC, ICC values were calculated using (1) β coefficients from the voxel-wise analysis, (2) contrasts from the voxel-wise analysis, (3) β coefficients from the ICA, and (4) contrasts from the ICA. All ICC calculations were completed in MATLAB R2019a using the “ICC” function (McGraw and Wong, 1996; Salarian, 2016) and type “1–1” or “ICC_case_1_1.” Voxel-wise ICC calculations generated an ICC value at every voxel for either each β coefficient or each contrast. ICA ICC calculations generated an ICC value for each component for either each β coefficient or each contrast.

To test whether there was a relationship between degree of activation and ICC value, we conducted a Pearson correlation between ICC values and voxelwise activity. For this analysis, voxelwise activity was characterized in two ways: (1) the mean activation of that voxel (i.e., y-intercept of the LME), and (2) the LME effect of valence.

## Results

### Demographic Characteristics

See Table 1 for clinical and demographic characteristics of this sample.

**Table 1:** Participant demographic characteristics.

**Variable**	**Mean (SD)**
Sample	*N* = 23
Age (yrs)	13.78 (1.76)
Verbal IQ	92.48 (13.16)
Ethnicity	35% Caucasian
	57% African American
	9% Biracial
	0% Hispanic
PTSD	36.26 (19.04)
Assault type	Physical assault 35%
	Physical abuse 91%
	Sexual abuse 86%
# comorbid diangoses	2.83 (2.19)
Current depressive disorder	55%
Current anxiety disorder	73%
Child behavioral checklist	Anxious depressed 9.35 (5.78)
	Withdrawn depressed 6.00 (2.78)
	Somatic complaints 6.09 (5.29)
	Social problems 5.96 (4.92)
	Thought problems 6.22 (5.03)
	Attention problems 9.35 (5.39)
	Rule breaking problems 6.65 (6.87)
	Aggressive behavior 10.39 (6.55)

*PTSD symptom severity refers to baseline UCLA PTSD scores. Assault type was assessed using the NSA*.

### Regional Activation During Emotion Processing Task

**Figure 2:**
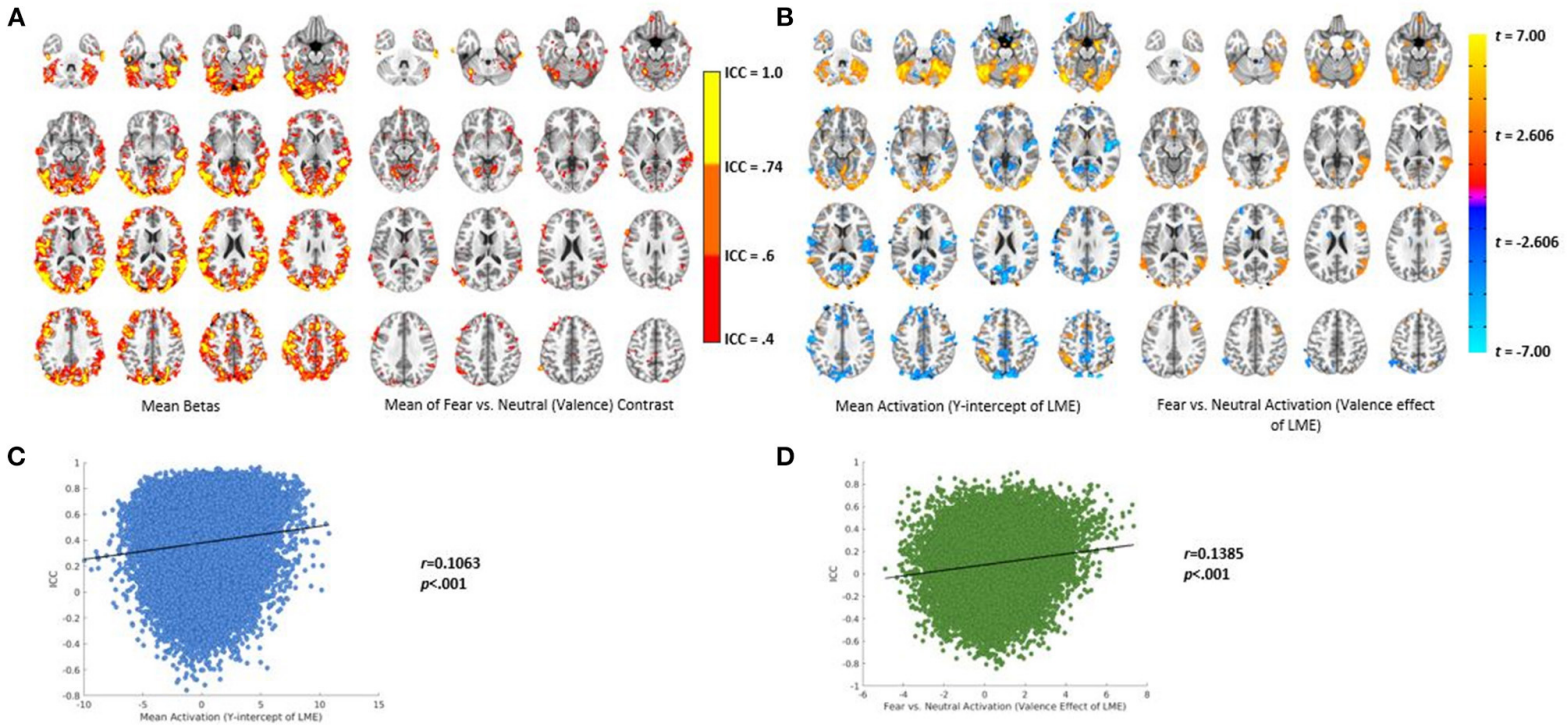
Results of the voxel-wise analysis and ICC calculation. **(A)** Left—ICC values using the mean β coefficient from the voxel-wise analysis. Right—ICC values using the mean valence contrast (fear vs. neutral) from the voxel-wise analysis. **(B)** Activation maps from the LMEMs. Left—mean activation (i.e., the Y-intercept). Right—activation for the valence contrast (fear vs. neutral). **(C)** Pearson correlation of the relationship between mean activation and ICC value using β coefficients from the voxel-wise analysis. **(D)** Pearson correlation of the relationship between the valence effect of the LMEMs and ICC values using the valence contrast (far vs. neutral) from the voxel-wise analysis.

Thirty Significant Clusters (*t* = 3.35, Corrected *p* < 0.05) Were Identified for the Mean Activation Throughout all Conditions of the Task, Including Bilateral Amygdala, Bilateral Fusiform Gyrus, and Bilateral Dorsal Anterior Cingulate. Six Significant Clusters Were Identified for the Valence Effect of the LMEM, Including the Right Amygdala, Left Caudate, and Right Temporal Pole. See Supplementary Tables 1, 2 for a Full List of the Significant Clusters; see Figure 2B for Activation Maps.

#### Voxelwise ICC Values

ICC values calculated with voxelwise mean β coefficients and the fear vs. neutral (valence) contrast are displayed in Figure 2A. As can be seen, different regions had varying degrees of reliability from poor to excellent.

### Degree of Activation and ICC Values

As can be seen in Figure 2C, we found a significant, small positive relationship between mean activation and ICC values calculated using β coefficients (*r* = 0.106, *p* < 0.001). There was also a significant, small positive relationship between the valence effect of the LMEM and ICC values calculated using contrasts (*r* = 0.138, *p* < 0.001 see Figure 2D).

### Network Activation During Emotion Processing Task

Seven of the nine components were significantly related to the task (i.e., main effect of valence, main effect of duration, valence × duration interaction). See Supplementary Table 3 for results of the LMEM.

#### Network ICC Values

**Figure 3:**
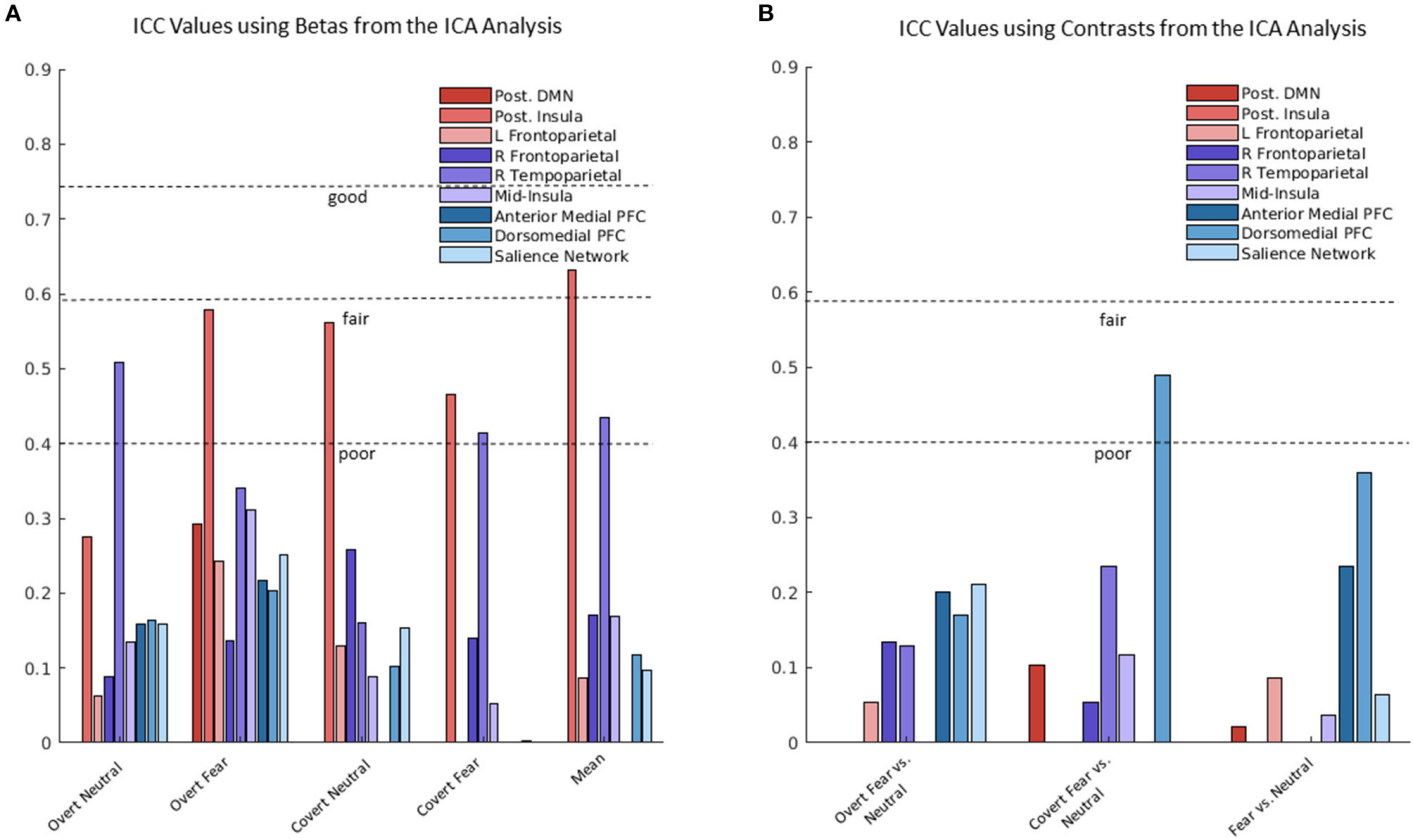
Intraclass correlation coefficient (ICC) values using β coefficients and contrasts from the independent components analysis (ICA) analysis. **(A)** Range of ICC values using betas for the nine ICA components (i.e., networks). **(B)** Range of ICC values using contrasts for the nine ICA components (i.e., networks).

ICC values from the ICA analysis were calculated using β coefficients and contrasts separately (see Figure 3). Two of the nine components reached a fair to good level of reliability when ICC values were calculated using β coefficients. One component with dominant loadings in the posterior insula reached a fair level of reliability for the overt fear (ICC = 0.579) and covert neutral (ICC = 0.561) conditions, as well as a good reliability for mean activation across the task (ICC = 0.632). The other component, with dominant loadings in the right temperoparietal region, reached a fair level of reliability for the overt neutral (ICC = 0.509), covert fear (ICC = 0.413), and mean activation (ICC = 0.434). Another component with dominant loadings in the dorsomedial PFC reached above poor reliability for the covert fear vs. covert neutral contrast (ICC = 0.490) when ICC values were calculated using contrasts.

## Discussion

Establishing good test-retest reliability of fMRI is crucial for drawing inferences about individual differences in neural activation, and is thus crucial for utilizing fMRI to neurologically characterize cognition and support research in clinical populations. The aim of the study was to methodologically assess analytical approaches and calculation of test-retest reliability of fMRI using ICC. Increased reliability has been found using predictive modeling on resting state data (Taxali et al., 2021), and through our work has been shown to apply to task-based fMRI as well. Furthermore, the importance of methodological decisions in reliability evaluation has been proven in clinical settings (Compère et al., 2020) and extended through this work. Overall, the present data shows that (1) for both the voxelwise analysis and ICA, use of β coefficients in ICC calculation, compared to contrasts, yielded greater ICC values, (2) there is a positive relationship between ICC values and degree of neural activation, and (3) fair to excellent ICC values are concentrated in cortical, rather than subcortical, regions.

Our finding of higher ICC values when using β coefficients compared to contrasts is unsurprising given the amounting literature concerning the unreliable and error-prone nature of difference scores (i.e., contrasts). First, two measurements observed at the same time point are not independent and highly correlated (Cronbach and Furby, 1970). Therefore, utilizing a change score results in a high degree of error. Second, difference scores have low between subject variability, and greater reliability is achieved with increased between subject variability (Zeynep Enkavi et al., 2019). As such, the use of a difference score in individual difference analyses, such as test-retest reliability, will likely lead to lower estimates of reliability. For some time, researchers have acknowledged these challenges with change scores. For example, within clinical research the majority of treatment studies do not assess effects of treatment based on changes between baseline and post-treatment, largely because the difference between pre- and post-treatment is sensitive to changes in variance (Vickers, 2001). To circumvent the use of differences scores in imaging research, approaches that directly analyze the β coefficients, such as ANOVAs or LMEMs, are preferable. Unlike a contrast score, which represents the difference in neural activation between two similar conditions, β coefficients directly characterize percent signal change in the blood-oxygen-level-dependent (BOLD) timecourses, which is a more direct measure of functional activation.

Our investigation found a small but positive relationship between ICC values and neural activation. This relationship suggests the impact of task activation magnitude on reliability of task activation is of only small effect. However, our finding is consistent with the existing reliability literature (Caceres et al., 2009; Bennett and Miller, 2013; Brandt et al., 2013; Holiga et al., 2018; Heckendorf et al., 2019; Bossier et al., 2020; Li et al., 2020; Korucuoglu et al., 2021). With task-based fMRI, certain regions are expected to be active depending on the task employed (e.g., amygdala activation during threat processing). By default, regions that are consistently activated in response to specific task paradigms should exhibit greater reliability. Our results also showed that higher ICC values were concentrated in cortical regions (see Figure 2A), which is consistent with a prior report of fMRI reliability (Korucuoglu et al., 2020). During a risk taking behavior task in a sample of monozygotic twins, cortical regions tended to have greater ICC values than subcortical regions (Korucuoglu et al., 2020). Similarly, our finding of higher ICC in the cortical regions compared to subcortical is expected given that the neural signal from cortical regions tends to be stronger than subcortical regions (Ojemann et al., 1997; Seitzman et al., 2020).

Additionally, we anticipated that there would be differences in reliability depending on the analysis used to characterize neural activation, specifically in favor of the network-level analysis. However, the data revealed an unexpected finding of higher max ICC values for the voxelwise analysis. Both the voxelwise analysis and ICA yielded a range of ICC values. The finding that more ICC values from the voxelwise analysis had excellent reliability compared to ICA could be explained by the fact that the voxelwise analysis likely reflects the mass univariate search across all voxels, thereby separately characterizing areas with high and low ICC. Reliability assessed from a voxelwise analysis results in an ICC value at each voxel independently. Alternatively, ICA by definition considers all voxels within a network that coactivate. As such, ICA reflects reliability of an entire network collapsed across many voxels, some of which may have high or low ICC. The observed differences in ICC values for the ICA and voxelwise analyses could also stem from one analysis being more or less sensitive to neural changes over time. In other words, voxelwise analyses may be less sensitive to neural changes associated with treatment, which could explain higher voxelwise ICC values compared to ICA ICC values. Additional research is needed to further examine the implications of voxelwise vs. network-level analyses when examining test-retest reliability.

Collectively, based on our findings that discrepancies in ICC values may depend on analytical approaches, we recommend that researchers must critically consider the objectives of their investigation to determine whether a voxelwise or network level analysis is warranted. For instance, given that cortical regions and active regions have higher ICC values, an ROI analysis will likely result in higher estimations of reliability. On the other hand, the maximum ICC values from a network level analysis will likely be lower because the analysis is not tuned to a specific set of voxels or single anatomical region.

The present study is not without limitations. First, with 23 participants, the sample size is relatively small. A larger sample size would lend to a more comprehensive assessment of methodological considerations when calculating test-retest reliability and greater statistical power (Bossier et al., 2020). Second, the data was taken from an adolescent treatment sample. One limitation of this sample is that adolescent movement in the scanner could have influenced the results (i.e., the mean number of TRs was 83 and 76% at time one and time two, respectively). Second of all, neural activity was expected to change from pre- to post-treatment; therefore, reliability estimates were conservative. Similarly, habituation of neural activity to task stimuli (e.g., faces) is expected to occur in a healthy population (Breiter et al., 1996; Fischer et al., 2003; Plichta et al., 2014) but was not accounted for in the present study. In future studies, this limitation could be remedied by including a control group that would serve as a comparison, which could account for habituation to the task and provide a more accurate estimate of reliability across the timeframe. Interpretation of the results may be skewed due to the lack of a healthy control group. Alternatively, the investigation into ICC calculation using β coefficients vs. contrasts should be reexamined in a non-treatment sample. Additionally, the sample consisted of adolescent females still going through development, which could result in lower reliability estimates. However, previous work has investigated reliability in children and found neural activity to be reliable (Song et al., 2012; Somandepalli et al., 2015). Despite these limitations, this investigation was still able to compare different analytical approaches to brain data analyses and ICC calculation. Third, although commonly administered, the emotion processing task employed is a passive, rather than active task, which does not necessitate strong neural engagement outside of visual and motor cortex. A task that is substantially cognitively demanding would enhance the neural signal and activation of regions involved in higher order cognition. Given the relationship between ICC values and degree of activation present in the literature and found here, an active task would likely yield higher reliability estimates.

To our knowledge, this is the first study to investigate the use of β coefficients vs. contrasts and voxelwise vs. network-level analyses in the assessment of fMRI test-retest reliability. When assessing reliability of fMRI, our results support the use of β coefficients rather than contrasts when calculating ICC. Specifically, we found that ICC calculation using β coefficients from the voxelwise analysis and ICA yielded higher ICC values compared to contrasts across the brain. Previous reports of low test-retest reliability of fMRI may be attributable to methodological considerations when analyzing brain data and calculating reliability estimates. The findings presented here enhance our understanding of test-retest reliability and support the fact that methodological considerations (e.g., data analysis procedure, measure of neural activity) have a profound influence on reliability estimates.

## Data Availability Statement

The raw data supporting the conclusions of this article will be made available by the authors, without undue reservation.

## Ethics Statement

The studies involving human participants were reviewed and approved by University of Arkansas for Medical Sciences Institutional Review Board. The patients/participants provided their written informed consent to participate in this study.

## Author Contributions

MH led the analysis of the data, drafted and critically revised the work for publication, provided final approval for the version to be published, provided a substantial contribution to the study conception, data analysis, manuscript drafting and intellectual content, and provided final approval of the version to be published. KC contributed to drafting the manuscript and provided meaningful feedback on the tables and figures and theoretical considerations in the interpretation of results. JC led the conception and design of the project, led the acquisition and pre-processing of the neuroimaging data, contributed to the interpretation of the results for the manuscript, and provided feedback and critical revisions to the manuscript for intellectual and theoretical content. All authors contributed meaningfully to the preparation of this manuscript. All authors contributed to the article and approved the submitted version.

## Funding

Portions of this work were funded by the National Institutes of Mental Health (Grant No. MH106860) and the Brain and Behavior Research Foundation.

## Conflict of Interest

The authors declare that the research was conducted in the absence of any commercial or financial relationships that could be construed as a potential conflict of interest.

## Publisher's Note

All claims expressed in this article are solely those of the authors and do not necessarily represent those of their affiliated organizations, or those of the publisher, the editors and the reviewers. Any product that may be evaluated in this article, or claim that may be made by its manufacturer, is not guaranteed or endorsed by the publisher.

## References

[B1] Bennett C. M. Miller M. B. (2013). FMRI reliability: influences of task and experimental design. Cogn. Affect. Behav. Neurosci. 13, 690–702. 10.3758/s13415-013-0195-1 23934630

[B2] Bernstein D. P. Fink L. Handelsman L. Foote J. M. Lovejoy Wenzel K.. (1994). Initial reliability and validity of a new retrospective measure of child abuse and neglect. Am. J. Psychiatry 151, 1132-1136. 10.1176/ajp.151.8.1132 8037246

[B3] Blautzik J. Keeser D. Berman A. Paolini M. Kirsch V. Mueller S.. (2013). Long-Term test-retest reliability of resting-state networks in healthy elderly subjects and patients with amnestic mild cognitive impairment. J. Alzheimers Dis. 34, 741–754. 10.3233/JAD-111970 23271315

[B4] Bossier H. Roels S. P. Seurinck R. Banaschewski T. Barker G. J. Bokde A. L. W.. (2020). The empirical replicability of task-based FMRI as a function of sample size. NeuroImage 212, 1–12. 10.1016/j.neuroimage.2020.116601 32036019

[B5] Brandt D. J. Sommer J. Krach S. Bedenbender J. Kircher T. Paulus F. M.. (2013). Test-Retest reliability of FMRI brain activity during memory encoding. Front. Psychiatry 4, 163. 10.3389/fpsyt.2013.00163 24367338 PMC3856399

[B6] Breiter H. C. Etcoff N. L. Whalen P. J. Kennedy W. A. Rauch S. L. Buckner R. L.. (1996). Response and habituation of the human amygdala during visual processing of facial expression. Neuron 17, 875–887. 10.1016/S0896-6273(00)80219-6 8938120

[B7] Brunetti M. G. Sepede G. Mingoia C. Catani A. Ferretti A. Merla C.. (2010). Elevated response of human amygdala to neutral stimuli in mild post traumatic stress disorder: neural correlates of generalized emotional response. Neuroscience 168, 670–679. 10.1016/j.neuroscience.2010.04.024 20416363

[B8] Caceres A. Hall D. L. Zelaya F. O. Williams S. C. R. Mehta M. A. (2009). Measuring FMRI reliability with the intra-class correlation coefficient. NeuroImage 45, 758–768. 10.1016/j.neuroimage.2008.12.035 19166942

[B9] Calhoun V. D. Adali T. Pearlson G. D. Pekar J. J. (2002). A method for making group inferences from functional mri data using independent component analysis. Hum. Brain Map. 16, 131. 10.1002/hbm.10044 11559959 PMC6871952

[B10] Chou Y. H. Panych L. P. Dickey C. C. Petrella J. R. Chen N. K. (2012). Investigation of long-term reproducibility of intrinsic connectivity network mapping: a resting-state FMRI study. Am. J. Neuroradiol. 33, 833–838. 10.3174/ajnr.A2894 22268094 PMC3584561

[B11] Cicchetti D. V. (1993). Guidelines, criteria, and rules of thumb for evaluating normed and standardized assessment instruments in psychology. Psychol. Assess. 6, 284–290. 10.1037/1040-3590.6.4.284

[B12] Cisler J. M. Esbensen K. Sellnow K. Ross M. Weaver S. Sartin-Tarm A.. (2019). Differential roles of the salience network during prediction error encoding and facial emotion processing among female adolescent assault victims. Biol. Psychiatry Cogn. Neurosci. Neuroimaging 4, 371–380. 10.1016/j.bpsc.2018.08.014 30343131 PMC6638574

[B13] Cisler J. M. Privratsky A. Smitherman S. Herringa R. J. Kilts C. D. (2018). Large-Scale brain organization during facial emotion processing as a function of early life trauma among adolescent girls. NeuroImage Clin. 17, 778–785. 10.1016/j.nicl.2017.12.001 29527485 PMC5842665

[B14] Cisler J. M. Sigel B. A. Kramer T. L. Smitherman S. Karin V. Pemberton J.. (2015). Amygdala response predicts trajectory of symptom reduction during trauma-focused cognitive-behavioral therapy among adolescent girls with PTSD. J. Psychiatr. Res. 71, 33–40. 10.1016/j.jpsychires.2015.09.011 26522869 PMC4826076

[B15] Cisler J. M. Sigel B. A. Steele J. S. Smitherman S. Vanderzee K. Pemberton J.. (2016). Changes in functional connectivity of the amygdala during cognitive reappraisal predict symptom reduction during trauma-focused cognitive-behavioral therapy among adolescent girls with post-traumatic stress disorder. Psychol. Med. 46, 3013–3023. 10.1017/S0033291716001847 27524285

[B16] Cole D. M. Smith S. M. Beckmann C. F. (2010). Advances and pitfalls in the analysis and interpretation of resting-state FMRI data. Front. Syst. Neurosci. 4, 8. 10.3389/fnsys.2010.00008 20407579 PMC2854531

[B17] Compère L. Siegle G. J. Young K. (2020). Importance of test-retest reliability for promoting fMRI based screening and interventions in major depressive disorder. BioRxiv [Preprint]. 10.1101/2020.12.11.421750 34247184 PMC8272717

[B18] Cronbach L. J. Furby L. (1970). How we should measure ‘change': or should we?” Psychol. Bull. 74, 68–80. 10.1037/h0029382

[B19] Eklund A. Nichols T. E. Knutsson H. (2016). “Erratum: Cluster failure: Why fMRI inferences for spatial extent have inflated false-positive rates,” in Proceedings of the National Academy of Sciences of the United States of America, Vol. 113, E4929. 10.1073/pnas.1612033113 27357684 PMC4948312

[B20] Elliott M. L. Knodt A. R. Ireland D. Morris M. L. Poulton R. Ramrakha S.. (2020). What is the test-retest reliability of common task-functional MRI measures? New empirical evidence and a meta-analysis. Psychol. Sci. 31, 792–806. 10.1177/0956797620916786 32489141 PMC7370246

[B21] Fischer H. Wright C. I. Whalen P. J. McInerney S. C. Shin L. M. Rauch S. L. (2003). Brain habituation during repeated exposure to fearful and neutral faces: a functional MRI study. Brain Res. Bull. 59, 387–392. 10.1016/S0361-9230(02)00940-1 12507690

[B22] Fröhner J. H. Teckentrup V. Smolka M. N. Kroemer N. B. (2019). Addressing the reliability fallacy in FMRI: similar group effects may arise from unreliable individual effects. NeuroImage 195, 174–189. 10.1016/j.neuroimage.2019.03.053 30930312

[B23] Gratz K. L. Roemer L. (2004). multidimensional assessment of emotion regulation and dysregulation: Development, factor structure, and initial validation of the difficulties in emotion regulation scale. J. Psychopathol. Behav. Assess. 26, 41–54. 10.1023/B:JOBA.0000007455.08539.9434775912

[B24] Guo C. C. Kurth F. Zhou J. Mayer E. A. Eickhoff S. B. Kramer J. H.. (2012). One-Year test-retest reliability of intrinsic connectivity network FMRI in older adults. NeuroImage 61, 1471–1483. 10.1016/j.neuroimage.2012.03.027 22446491 PMC4226138

[B25] Heckendorf E. Bakermans-Kranenburg M. J. van Ijzendoorn M. H. Huffmeijer R. (2019). Neural responses to children's faces: test–retest reliability of structural and functional MRI. Brain Behav. 9, e01192. 10.1002/brb3.1192 30739395 PMC6422824

[B26] Holiga Š. Sambataro F. Luzy C. Greig G. Sarkar N. Renken R. J.. (2018). Test-Retest reliability of task-based and resting-state blood oxygen level dependence and cerebral blood flow measures. PLoS ONE 13, e0206583. 10.1371/journal.pone.0206583 30408072 PMC6224062

[B27] Infantolino Z. P. Luking K. R. Sauder C. L. Curtin J. J. Hajcak G. (2018). Robust is not necessarily reliable: from within-subjects FMRI contrasts to between-subjects comparisons. NeuroImage 173, 146–152. 10.1016/j.neuroimage.2018.02.024 29458188 PMC5912348

[B28] Kaufman J. Birmaher B. Brent D. Rao U. Flynn C. Moreci P.. (1997). Schedule for affective disorders and schizophrenia for school-age children-present and lifetime version (K-SADS-PL): Initial reliability and validity data. J. Am. Acad. Child. Adoles. 36, 980-988. 10.1097/00004583-199707000-00021 9204677

[B29] Kilpatrick D. G. Acierno R. Saunders B. Resnick H. S. Best C. L. Schnurr P. P. (2000). Risk factors for adolescent substance abuse and dependence: Data from a national sample. J. Consult. Clin. Psychol. 68, 19–30. 10.1037/0022-006x.68.1.19 10710837

[B30] Koo T. K. Li M. Y. (2016). A guideline of selecting and reporting intraclass correlation coefficients for reliability research. J. Chiroprac. Med. 15, 155–163. 10.1016/j.jcm.2016.02.012 27330520 PMC4913118

[B31] Korucuoglu O. Harms M. P. Astafiev S. V. Golosheykin S. Kennedy J. T. Barch D. M.. (2021). Test-Retest reliability of neural correlates of response inhibition and error monitoring: an FMRI study of a stop-signal task. Front. Neurosci. 15, 624911. 10.3389/fnins.2021.624911 33584190 PMC7875883

[B32] Korucuoglu O. Harms M. P. Astafiev S. V. Kennedy J. T. Golosheykin S. Barch D. M.. (2020). Test-Retest reliability of FMRI-measured brain activity during decision making under risk. NeuroImage 214, 116759. 10.1016/j.neuroimage.2020.116759 32205253 PMC7846028

[B33] Li X. Pan Y. Fang Z. Lei H. Zhang X. Shi H.. (2020). Test-Retest reliability of brain responses to risk-taking during the balloon analogue risk task. NeuroImage 209, 116495. 10.1016/j.neuroimage.2019.116495 31887425 PMC7061333

[B34] McDermott T. J. Kirlic N. Akeman E. Touthang J. Cosgrove K. T. DeVille D. C.. (2020). Visual cortical regions show sufficient test-retest reliability while salience regions are unreliable during emotional face processing. NeuroImage 220, 117077. 10.1016/j.neuroimage.2020.117077 32574806 PMC7875460

[B35] McGraw K. O. Wong S. P. (1996). Forming inferences about some intraclass correlation coefficients 1, 30. 10.1037/1082-989X.1.1.30

[B36] Menon V. (2011). Large-Scale brain networks and psychopathology: a unifying triple network model. Trends Cogn. Sci. 15, 483–506. 10.1016/j.tics.2011.08.003 21908230

[B37] Morales C. Gohel S. Li X. Scheiman M. Biswal B. B. Santos E. M.. (2020). Test–Retest reliability of functional magnetic resonance imaging activation for a vergence eye movement task. Neurosci. Bull. 36, 506–518. 10.1007/s12264-019-00455-9 31872328 PMC7186292

[B38] Noble S. Scheinost D. Constable R. T. (2019). A decade of test-retest reliability of functional connectivity: a systematic review and meta-analysis. NeuroImage 203, 116157. 10.1016/j.neuroimage.2019.116157 31494250 PMC6907736

[B39] Ojemann J. G. Akbudak E. Snyder A. Z. McKinstry R. C. Raichle M. E. Conturo T. E. (1997). Anatomic localization and quantitative analysis of gradient refocused echo-planar FMRI susceptibility artifacts. NeuroImage 6, 156–167. 10.1006/nimg.1997.0289 9344820

[B40] Plichta M. M. Grimm O. Morgen K. Mier D. Sauer C. Haddad L.. (2014). Amygdala habituation: a reliable FMRI phenotype. NeuroImage 103, 383–390. 10.1016/j.neuroimage.2014.09.059 25284303

[B41] Plichta M. M. Schwarz A. J. Grimm O. Morgen K. Mier D. Haddad L.. (2012). Test-Retest reliability of evoked BOLD signals from a cognitive-emotive FMRI test battery. NeuroImage 60, 1746–1758. 10.1016/j.neuroimage.2012.01.129 22330316

[B42] Power J. D. Mitra A. Laumann T. O. Snyder A. Z. Schlaggar B. L. Petersen S. E. (2014). Methods to detect, characterize, and remove motion artifact in resting state fMRI. NeuroImage 84, 320-341. 10.1016/j.neuroimage.2013.08.048 23994314 PMC3849338

[B43] Raemaekers M. Vink M. Zandbelt B. van Wezel R. J. A. Kahn R. S. Ramsey N. F. (2007). Test-Retest reliability of FMRI activation during prosaccades and antisaccades. NeuroImage 36, 532–542. 10.1016/j.neuroimage.2007.03.061 17499525

[B44] Rauch S. L. Whalen P. J. Shin L. M. McInerney S. C. MacKlin M. L. Lasko N. B.. (2000). Exaggerated amygdala response to masked facial stimuli in posttraumatic stress disorder: a functional MRI study. Biol. Psychiatry 47, 769–776. 10.1016/S0006-3223(00)00828-3 10812035

[B45] Ross M. C. Cisler J. M. (2020). Altered large-scale functional brain organization in posttraumatic stress disorder: a comprehensive review of univariate and network-level neurocircuitry models of PTSD. NeuroImage: Clinical 27:102319. 10.1016/j.nicl.2020.102319 32622316 PMC7334481

[B46] Salarian A. (2016). Intraclass Correlation Coefficient (ICC). MathWorks. Available online at: https://www.mathworks.com/matlabcentral/fileexchange/22099-intraclass-correlation-coefficient-icc (accessed November 22, 2021).

[B47] Seitzman B. A. Gratton C. Marek S. Raut R. V. Dosenbach N. U. F. Schlaggar B. L.. (2020). A set of functionally-defined brain regions with improved representation of the subcortex and cerebellum. NeuroImage 206, 116290. 10.1016/j.neuroimage.2019.116290 31634545 PMC6981071

[B48] Sharp C. Goodyer I. M. Croudace T. J. (2006). The short mood and feelings questionnaire (SMFQ): A unidimensional item response theory and categorical data factor analysis of self-report ratings from a community sample of 7-through 11-year-old children. J. Abnorm. Child Psychol. 34, 365-377. 10.1007/s10802-006-9027-x 16649000

[B49] Sheehan D. V. Sheehan K. H. Shytle R. D. Janavs J. Bannon Y. Rogers J. E.. (2010). Reliability and validity of the mini international neuropsychiatric interview for children and adolescents (MINI-KID). J. Clin. Psychiatry 71:17393. 10.4088/JCP.09m05305whi 20331933

[B50] Siegel J. S. Power J. D. Dubis J. W. Vogel A. C. Church J. A. Schlaggar B. L.. (2014). Statistical improvements in functional magnetic resonance imaging analyses produced by censoring high-motion data points. Human Brain Mapp. 35, 1981–1996. 10.1002/hbm.22307 23861343 PMC3895106

[B51] Somandepalli K. Kelly C. Reiss P. T. Zuo X. Craddock R. C. Yan C.. (2015). Short-Term test–retest reliability of resting state FMRI metrics in children with and without attention-deficit/hyperactivity disorder. Dev. Cogn. Neurosci. 15, 83–93. 10.1016/j.dcn.2015.08.003 26365788 PMC6989828

[B52] Song J. Desphande A. S. Meier T. B. Tudorascu D. L. Vergun S. Nair V. A.. (2012). Age-Related differences in test-retest reliability in resting-state brain functional connectivity. PLoS ONE 7, e49847. 10.1371/journal.pone.0049847 23227153 PMC3515585

[B53] Steinber A. M. Brymer M. J. Decker K. B. Pynoos R. S. (2004). The university of california at los angeles post-traumatic stress disorder reaction index. Curr. Psy. 6, 96–100. 10.1007/s11920-004-0048-2 15038911

[B54] Steinberg A. M. Brymer M. J. Kim S. Briggs E. C. Ippen C. G. Ostrowski S. A.. (2013). Psychometric properties of the UCLA PTSD reaction index: Part I. J. Traum. Stress. 26, 1–9. 10.1002/jts.21780 23417873

[B55] Taxali A. Angstadt M. Rutherford S. Sripada C. (2021). Boost in test-retest reliability in resting state fmri with predictive modeling. Cereb. Cortex 31, 2822-2833. 10.1093/cercor/bhaa390 33447841 PMC8599720

[B56] Vickers A. (2001). The use of percentage change from baseline as an outcome in a controlled trial is statistically inefficient: a simulation study. Hypertension 1, 1–4. 10.1186/1471-2288-1-6 11459516 PMC34605

[B57] Williams L. M. Liddell B. L. Kemp A. H. Bryant R. A. Meares R. A. Peduto A. S.. (2006). Amygdala-Prefrontal dissociation of subliminal and supraliminal fear. Hum. Brain Mapp. 27, 652–661. 10.1002/hbm.20208 16281289 PMC6871444

[B58] Zeynep Enkavi A. Eisenberg I. W. Bissett P. G. Mazza G. L. MacKinnon D. P. Marsch L. A.. (2019). Large-Scale analysis of test–retest reliabilities of self-regulation measures. Proc. Natl. Acad. Sci. U.S.A. 116, 5472–5477. 10.1073/pnas.1818430116 30842284 PMC6431228

