# Supplementary material for: Test-Retest Reliability of fMRI During an Emotion Processing Task: Investigating the Impact of Analytical Approaches on ICC Values
Source: Front Neuroimaging. 2022 May 10;1:859792. doi: 10.3389/fnimg.2022.859792 (PMC9245148; doi:10.3389/fnimg.2022.859792)
Supplement: Supplementary file 1 [file Data_Sheet_1.docx]

# Supplementary Tables

| **Supplemental Table 1.** Significant clusters of activation (p < .05 corrected for multiple comparisons) for the mean activation. | | | | | |
| --- | --- | --- | --- | --- | --- |
| **Region** | **MNI center-of-mass coordinates** | | | **Peak *t*** | **Cluster size** |
|  | **X** | **Y** | **Z** |  |  |
| R Ventral Visual/ Fusiform Gyrus | -28.2 | 66.9 | -16.3 | 10.14 | 1289 |
| L Cuneus | 1.0 | 72.1 | 25.1 | -9.94 | 699 |
| L Ventral Visual/ Fusiform Gyrus | 36.9 | 69.6 | -12.0 | 10.51 | 631 |
| R Posterior Insula | -45.9 | 14.2 | 9.7 | -8.23 | 250 |
| L Primary Motor Cortex | 39.4 | 19.2 | 61.9 | 6.68 | 225 |
| L Precuneus | 1.7 | 46.7 | 50.9 | -6.39 | 190 |
| R Premotor Cortex | -52.4 | 7.7 | 36.3 | -7.78 | 152 |
| L Premotor Cortex | 61.3 | 8.7 | 25.1 | -7.00 | 149 |
| L Intraparietal | 39.2 | 49.7 | 47.2 | 8.40 | 116 |
| R Amygdala | -20.5 | 5.7 | -14.7 | 10.22 | 65 |
| R Temporal Pole | -39.4 | -19.2 | -30.0 | 8.37 | 54 |
| L Amygdala | 21.7 | 6.8 | -15.5 | 10.79 | 44 |
| L Anterior Middle Frontal Gyrus | 27.9 | -50.3 | 33.6 | -5.52 | 36 |
| L Inferior Frontal Gyrus | 54.9 | -22.1 | 3.4 | -5.03 | 35 |
| L Middle Frontal Gyrus | 45.5 | -25.1 | 21.4 | 5.89 | 31 |
| L Cerebellum/Parahippocampal | 21.9 | 39.5 | -20.6 | 5.97 | 30 |
| R Superior Parietal | -19.5 | 51.7 | 69.2 | -5.94 | 30 |
| L Dorsal Anterior Cingulate | 8.8 | -29.7 | 32.4 | -5.82 | 28 |
| R Anterior Temporal Pole | -46.7 | -3.7 | -36.9 | 6.05 | 26 |
| L Precentral Gyrus | 32.0 | 8.9 | 51.4 | 5.66 | 26 |
| R Dorsomedial PFC | -4.8 | -43.9 | 37.0 | -5.47 | 25 |
| R Mid-Cingulate | -6.6 | 3.0 | 46.1 | -4.74 | 25 |
| L Supplementary Motor | 17.5 | -2.4 | 71.2 | 5.00 | 23 |
| L Parietal Lobule | 57.5 | 39.6 | 28.5 | -5.54 | 23 |
| L Pre SMA | 6.7 | -1.8 | 62.8 | 6.08 | 22 |
| R Anterior Temporal Pole | -55.9 | -2.5 | -24.4 | -6.45 | 22 |
| L Medial Temporal Pole | 38.9 | -5.6 | -26.1 | 8.00 | 21 |
| L Medial Prefrontal/Orbitofrontal | 8.3 | -60.4 | -7.9 | 5.29 | 21 |
| R Dorsal Anterior Cingulate | -7.8 | -23.6 | 32.4 | -5.27 | 21 |
| L Middle Temporal Gyrus | 47.8 | 67.9 | 6.9 | 5.91 | 17 |
| L Tempoparietal Junction | 45.4 | 59.5 | 24.2 | -6.54 | 17 |

| **Supplemental Table 2.** Significant clusters of activation (p < .05 corrected for multiple comparisons) for the main effect of valence. | | | | | |
| --- | --- | --- | --- | --- | --- |
| **Region** | **MNI center-of-mass coordinates** | | | **Peak *t*** | **Cluster size** |
|  | **X** | **Y** | **Z** |  |  |
| R Fusiform Gyrus | -46.7 | 54.8 | -7.6 | 7.34 | 483 |
| R Inferior Frontal | -48.8 | -24 | 18.3 | 5.52 | 153 |
| L Fusiform Gyrus | 45.8 | 60.8 | -16.0 | 6.20 | 117 |
| R Amygdala | -23.4 | 5.5 | -17.3 | 5.65 | 35 |
| L Caudate | 16.3 | -6.3 | 20.4 | -4.89 | 21 |
| R Temporal Pole | -60.1 | 1.2 | -19.0 | 4.52 | 18 |

| **Supplemental Table 3.** ICA LMEM results | | | | | | |
| --- | --- | --- | --- | --- | --- | --- |
| **Component** | **Main effect of valence** | | **Main effect of duration** | | **Valence x duration interaction** | |
|  | **T-statistic** | **P-Value** | **T-statistic** | **P-Value** | **T-statistic** | **P-Value** |
| **Posterior DMN** | -0.040 | 0.968 | **-4.581** | **<.001** | **-2.884** | **<.001** |
| **Posterior insula** | 0.154 | 0.877 | **-5.408** | **<.001** | -0.274 | 0.783 |
| **Left Frontoparietal** | **-5.106** | **<.001** | -0.725 | 0.469 | 0.207 | 0.836 |
| **Right frontoparietal** | 1.667 | 0.097 | **3.224** | **0.002** | 1.879 | 0.062 |
| **Right tempo/parietal** | **5.350** | **<.001** | **5.808** | **<.001** | 0.529 | 0.597 |
| **Mid-insula** | -0.248 | 0.804 | -0.149 | 0.882 | **3.219** | **0.002** |
| **Anterior medial PFC** | -0.172 | 0.863 | -0.485 | 0.628 | 0.734 | 0.464 |
| **Dorsomedial PFC** | 0.290 | 0.772 | -1.526 | 0.129 | -0.397 | 0.691 |
| **Salience network** | -1.710 | 0.089 | **4.760** | **<.001** | **3.661** | **<.001** |
|  |  |  |  |  |  |  |
| Note. All components with at least one significant main effect of valence or duration or a significant interaction between valence and duration were carried forward to reliability analyses. Of the three components that were not significant (anterior medial PFC, dorsomedial PFC, and insula), the anterior medial PFC and dorsomedial PFC components were carried forward. The anterior/medial PFC component was not carried forward, despite being significant. | | | | | | |
|  |  |  |  |  |  |  |
|  |  |  |  |  |  |  |
|  |  |  |  |  |  |  |
